# Impact of vector richness on the risk of vector‐borne disease: The role of vector competence

**DOI:** 10.1002/ece3.11082

**Published:** 2024-03-01

**Authors:** Lifan Chen, Zhiying Tan, Ping Kong, Yanli Zhou, Liang Zhou

**Affiliations:** ^1^ School of Arts and Sciences Shanghai University of Medicine and Health Sciences Shanghai China; ^2^ School of Health Science and Engineering University of Shanghai for Science and Technology Shanghai China; ^3^ Collaborative Innovation Center for Biomedicine Shanghai University of Medicine and Health Sciences Shanghai China

**Keywords:** amplification effect, dilution effect, diversity–disease relationship, epidemiology model, multi‐vector

## Abstract

A central goal of disease ecology is to identify the factors that drive the spread of infectious diseases. Changes in vector richness can have complex effects on disease risk, but little is known about the role of vector competence in the relationship between vector richness and disease risk. In this study, we firstly investigated the combined effects of vector competence, interspecific competition, and feeding interference on disease risk through a two‐vector, one‐host SIR‐SI model, and obtained threshold conditions for the occurrence of dilution and amplification effects. Secondly, we extended the above model to the case of *N* vectors and assumed that all vectors were homogeneous to obtain analytic expressions for disease risk. It was found that in the two‐vector model, disease risk declined more rapidly as interspecific competition of the high‐competence vector increased. When vector richness increases, the positive effects of adding a high‐competence vector species on disease transmission may outweigh the negative effects of feeding interference due to increased vector richness, making an amplification effect more likely to occur. While the addition of a highly competitive vector species may exacerbate the negative effects of feeding interference, making a dilution effect more likely to occur. In the *N*‐vector model, the effect of increased vector richness on disease risk was fully driven by the strength of feeding interference and interspecific competition, and changes in vector competence only quantitatively but not qualitatively altered the vector richness–disease risk relationship. This work clarifies the role of vector competence in the relationship between vector richness and disease risk and provides a new perspective for studying the diversity–disease relationship. It also provides theoretical guidance for vector management and disease prevention strategies.

## Graphical abstract

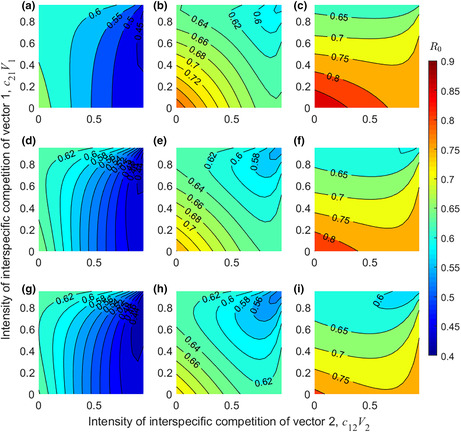

## 1 INTRODUCTION

Vector‐borne diseases contribute to a considerable fraction of all infectious diseases and pose a serious danger to both public health and wildlife management (Jones et al., 2008; Miller & Huppert, 2013). According to estimates from the World Health Organization, 1.5 million people per year pass away from vector‐borne diseases (WHO, 2004). With the rapid loss of global biodiversity and the emergence of new infectious diseases, understanding the key factors influencing the outbreak and spread of vector‐borne diseases is crucial for disease prevention and management.

The transmission of many vector‐borne diseases (e.g., Chagas disease, Lyme disease, and malaria) involves multiple hosts and vectors, which comprise extremely complex transmission networks. Changes in host or vector diversity may therefore directly or indirectly affect disease outbreaks and spread (Johnson et al., 2015). Several recent studies have shown that host diversity has a dilution effect on the risk of infectious disease (i.e., higher host diversity reduces disease risk), including for animal diseases (Johnson et al., 2013), plant diseases (Lacroix et al., 2014; Liu et al., 2016), and zoonotic diseases (Kilpatrick et al., 2006; Ostfeld & Keesing, 2000). However, the generality of the dilution effect remains controversial. For example, several recent theoretical and empirical studies have suggested that the diversity–disease relationship may be strongly context‐dependent (Cortez & Duffy, 2021; Liu et al., 2020). Several studies have found no significant effect (Salkeld et al., 2013; Vadell et al., 2020) or even an amplification effect (i.e., lower host diversity increases disease risk) in some natural ecosystems (Fletcher W Halliday et al., 2017; Wood et al., 2014). Furthermore, how disease risk is defined and measured can also lead to inconsistent results in diversity–disease relationship (Huang et al., 2015; Roberts & Heesterbeek, 2018). Considering these debates, identifying the key factors that influence the diversity–disease relationship and predicting the conditions under which dilution or amplification effects occur are necessary for disease prediction and control (Halliday et al., 2020; Rosenthal et al., 2021).

While substantial progress has been made in understanding the impact of host richness on disease risk (Civitello et al., 2015; Huang et al., 2016; Keesing & Ostfeld, 2021; Liu et al., 2022), vector richness has rarely been investigated (but see Hoi et al. (2020); Park et al. (2015); Takimoto et al. (2022)). In fact, many vector‐borne diseases are transmitted between hosts via multiple vectors. For instance, malaria has more than four mosquito vectors (Hoi et al., 2020, 2022), West Nile virus has 7–16 mosquito vectors (Roche et al., 2013), and Chagas disease has up to 10 triatomine vectors (Eduardo et al., 2018). Although vector richness may strongly influence the pathogen transmission (Brooks & Zhang, 2010), the effect of vector richness on disease risk is poorly understood.

Several empirical and theoretical studies have suggested that increasing vector richness is likely to amplify disease risk (Brooks & Zhang, 2010; Park et al., 2015; Roche et al., 2013). However, an increase in vector richness may inhibit pathogen transmission by interfering with effective vector–host contact (Tatchell, 1987). Recently, Takimoto et al. (2022) formulated a modeling work with interspecific competition and feeding interference among mosquitos to explore how vector species richness affects disease risk. They found vector richness can both amplified and diluted disease risk, depending on the combined effects of vector interspecific competition and feeding interference. This finding challenges previous conclusions about the positive correlation between vector richness and disease risk and has important implications for better understanding the relationship between diversity and disease risk. However, they did not take into account the role of vector competence (the ability of a vector to become infected with and transmit pathogens (Kain et al., 2022)).

Empirical evidence suggests that vector competence differs considerably among vector species. For example, in a synthesis study of Australian mosquitoes, by analyzing 68 laboratory studies of 111 mosquito‐virus pairs, researchers found that even within a genus, there was a significant difference in vector competence (Kain et al., 2022). Similarly, for Lyme disease, the pathogen is transmitted primarily through *Ixodes scapularis* and *Ixodes pacificus*, with the former having higher pathogen transmission efficiency than the latter (Couper et al., 2020). In an assessment of the vector competence to transmit Zika virus (ZIKV) in California, *Culex quinquefasciatus* was incompetent to transmit ZIKV, whereas *Aedes aegypti* was highly competent to transmit ZIKV (Main et al., 2018). To the best of our knowledge, it is unclear how changes in vector competence affect pathogen transmission.

In summary, despite numerous studies have examined how host richness affects disease risk, the impact of vector richness on the risk of vector‐borne disease remains unclear. By developing an SIR‐SI epidemiological model containing multiple vectors, we explored the following questions: (1) How do vector competence, feeding interference, and vector interspecific competition interact to influence the risk of vector‐borne disease? (2) Under what circumstances do the amplification and dilution effects occur? (3) How community *R*_0_ varies with vector richness? (4) Whether and how vector competence influences the effect of vector richness on disease risk? This study will improve our understanding of vector‐borne disease dynamics and provide theoretical guidance for the development of disease control strategies.

## 2 MATERIALS AND METHODS

In this section, we started with a two‐vector, one‐host model and derived the basic reproduction number as a measure of disease risk. Next, we compared the basic reproduction number of the single‐vector model and the two‐vector model to obtain threshold conditions for dilution and amplification effects. Finally, we extended the model to the case of *N* vectors and assumed that all vectors were homogeneous to obtain analytic expressions for disease risk.

### 2.1 Two‐vector, one‐host model

Inspired by the classic vector‐borne disease model (Keeling & Rohani, 2008), we modeled pathogen transmission in a two‐vector, one‐host system in which vectors also experience interspecific competition and feeding interference. This model is a special case (i.e., *N* = 2) of Takimoto et al. (2022). Hosts were classified as susceptible, infected, or recovered according to their infection status, while vectors were classified as susceptible or infected. Pathogens could only be transmitted via contact between host and vector, not by vector‐to‐vector or host‐to‐host routes. The following equations represent the dynamics of each model component:

(1) $$\begin{array}{c} \frac{{\mathrm{dV}}_{S,i}}{\mathrm{dt}}=\lambda_{v,i}\left(1-c_{\mathrm{ij}}V_{j}\right)-\mu_{v,i}V_{S,i}-\frac{k_{\mathrm{vh},i}b_{i}}{V_{i}}\frac{H_{I}}{H}V_{S,i}\\ \frac{{\mathrm{dV}}_{I,i}}{\mathrm{dt}}=\frac{k_{\mathrm{vh},i}b_{i}}{V_{i}}\frac{H_{I}}{H}V_{S,i}-\mu_{v,i}V_{I,i}\\ \frac{{\mathrm{dH}}_{S}}{\mathrm{dt}}=\lambda_{h}-\mu_{h}H_{S}-\sum_{i=1}^{2}\frac{k_{\mathrm{hv},i}b_{i}}{H}\frac{V_{I,i}}{V_{i}}H_{S}\\ \frac{{\mathrm{dH}}_{I}}{\mathrm{dt}}=\sum_{i=1}^{2}\frac{k_{\mathrm{hv},i}b_{i}}{H}\frac{V_{I,i}}{V_{i}}H_{S}-\mu_{h}H_{I}-\gamma_{h}H_{I}\\ \frac{{\mathrm{dH}}_{R}}{\mathrm{dt}}=\gamma_{h}H_{I}-\mu_{h}H_{R} \end{array}\;i,j\in \left\{1,2\right\},i\neq j$$

Table 1 lists the model parameters and their descriptions. Vector disease dynamics are represented by the first two equations in (1). $V_{S,i}$ and $V_{I,i}$ denote the number of susceptible and infected individuals for vector *i*, and $V_{i}$ is the total number of vector *i* individuals ($V_{i}$ = $V_{S,i}$ + $V_{I,i}$). $\lambda_{v,i}$ is the recruitment rate of the vector *i* when there is no interspecific competition, and $\lambda_{v,i}\left(1-c_{\mathrm{ij}}V_{j}\right)$ is the recruitment rate of vector *i* when regulated by interspecific competition. The term $k_{\mathrm{vh},i}\left(b_{i}/V_{i}\right)\left(H_{I}/H\right)V_{S,i}$ reflects how many vectors change from susceptible to infected per unit of time: $k_{\mathrm{vh},i}$ is the per capita transmission efficiency from host to vector *i*, $b_{i}/V_{i}$ is the per capita vector *i*‐host contact rate, *H* is the total number of hosts, and $H_{I}/H$ denotes the prevalence of disease in the host population.

**TABLE 1:** Model parameters.

Parameter	Definition
*V*_s,*i*_	Number of susceptible individuals of vector *i*
*V*_I,*i*_	Number of infected individuals of vector *i*
*V*_*i*_	Total population size of vector *i*, $V_{i}$ = $V_{S,i}$ + $V_{I,i}$
*H*_s_	Number of susceptible host individuals
*H*_I_	Number of infected host individuals
*H*_R_	Number of recovered host individuals
*H*	Total population size of the host, H = $H_{S}$ + $H_{I}$ + $H_{R}$
*λ*_v,*i*_	Recruitment rate of vector *i*
*C*_*ij*_	Intensity of interspecific competition of vector *j* on *i* ($i,j\in \left\{1,2\right\},i\neq j$)
*μ*_v,*i*_	Per capita death rate of vector *i*
*b*_*i*_	The number of vector *i‐* host contacts per unit of time
*k*_vh,*i*_	Per capita transmission efficiency from host to vector *i*
*λ*_*h*_	Recruitment rate of the host
*μ*_h_	Per capita death rate of the host
*k*_hv,*i*_	Per capita transmission efficiency from vector *i* to host
*γ*_h_	Per capita recovery rate of the host
*σ*_v,*i*_	Per capita maximum feeding intensity of vector *i*
*π*_*ij*_	Feeding interference intensity of vector *j* on *i*
*g*_*i*_	The competence of vector *i*

The last three equations in (1) represent the host transmission dynamics. As there is only one host species in the community, there is no interspecific competition. $\lambda_{h}$ is the recruitment rate of the host species. $\sum_{i=1}^{2}k_{\mathrm{hv},i}\left(b_{i}/H\right)\left(V_{I,i}/V_{i}\right)H_{S}$ reflects how many new hosts are infected per unit time: $k_{\mathrm{hv},i}$ denotes the per capita transmission efficiency from vector *i* to the host, $b_{i}/H$ denotes the per host contact rate with vector *i*, and $V_{I,i}/V_{i}$ denotes the prevalence of disease in the vector *i* population.

In the classic Ross‐Macdonald model (Anderson & May, 1991), the number of host–vector contacts is formulated as $b=\sigma_{v}V$, where V is the vector density and $\sigma_{v}$ denotes vector's maximum per capita feeding intensity. This formula has been widely used to explain how malaria and other vector‐borne diseases spread (Ngwa & Shu, 2000), yet it only considers the case of a single vector. In fact, the presence of other vectors may reduce the duration of host attacks by existing vectors, thereby interfering vector feeding success, that is, the feeding interference (Kelly, 2001). Some observational evidence suggests that the success of vector feeding in some species is limited by the number of possible feeding locations in smaller hosts (e.g., little exposed skin) (Tatchell, 1987). For example, ticks only attack the featherless areas (around the beak and eyes) of the gray catbird (Brinkerhoff et al., 2011), and also in sandflies, which congregate on the furless snouts of the rock hyrax (Svobodová et al., 2006). Considering the interference effect of the added vector *j* on the contact between vector *i* and the host, we modified the above formula for *b* as:

(2) $$b_{i}=\sigma_{v,i}V_{i}\frac{V_{i}}{V_{i}+\pi_{\mathrm{ij}}V_{j}}=\frac{\sigma_{v,i}V_{i}}{1+\pi_{\mathrm{ij}}V_{j}/V_{i}},$$

so that the total number of vector *i*‐host contacts (i.e., *b*_*i*_) is determined by the populations of both vectors. Here, $\pi_{\mathrm{ij}}$ represents the feeding interference caused by vector *j* on vector *i*. According to Equation (2), if $\pi_{\mathrm{ij}}=0$ or $V_{j}=0$, then *b*_*i*_ reverts to the form used for a single vector species ($b_{i}=\sigma_{v,i}V_{i}$). However, if feeding interference ($\pi_{\mathrm{ij}}$) is strong, or if the density of vector *j* ($V_{j}$) is much higher than that of vector *i*, then the number of host–vector *i* contacts will be greatly diminished. Note that for contact rate *b*_*i*_, we used a simplified form that differed from that of Takimoto et al. (2022) to obtain some analytical results.

### 2.2 An indicator of disease risk

Here, we used the basic reproduction number, *R*_0_, as an indicator of disease risk (i.e., the risk of outbreaks after the introduction of an infectious agent). *R*_0_ quantifies the number of secondary infections caused by a single primary infection in an otherwise susceptible population. It has been widely used to estimate the severity of epidemic outbreaks and to quantify the potential for pathogen transmission (Chen & Zhou, 2015; Dobson, 2004). The larger the *R*_0_, the higher the disease risk.

The value of *R*_0_ is derived from the dominant eigenvalue of the next‐generation matrix (Mick G Roberts & Heesterbeek, 2013). In detail, we first linearized the equations for infected hosts and vectors at the disease‐free equilibrium point ($V_{1},V_{2},H$). Then, we derived the transmission rate matrix *F* and the transition matrix *V* to obtain the next‐generation matrix $K=-{\mathrm{FV}}^{-1}$. The dominant eigenvalue of *K* is the basic reproduction number, *R*_0_ (see Appendix S1 for calculations). Next, we obtained:

(3) $$R_{0}=\sqrt{\frac{k_{\mathrm{vh},1}k_{\mathrm{hv},1}b_{1}^{2}}{H\left(\mu_{h}+\gamma_{h}\right)V_{1}\mu_{v,1}}+\frac{k_{\mathrm{vh},2}k_{\mathrm{hv},2}b_{2}^{2}}{H\left(\mu_{h}+\gamma_{h}\right)V_{2}\mu_{v,2}}}$$

Let $g_{i}=\frac{k_{\mathrm{vh},i}k_{\mathrm{hv},i}}{\mu_{v,i}}$, then *g*_*i*_ represents the efficiency with which the vector *i* is infected by biting an infected host species ($k_{\mathrm{vh},i}$) and transmits pathogens to other susceptible hosts ($k_{\mathrm{hv},i}$) during infection ($\mu_{v,i}$), that is, the ability of vector *i* to become infected with and transmit pathogens. Therefore, we use this index as a measure of vector competence. The higher *g*_*i*_, the higher the competence of vector *i*. Substituting the expression of *g*_*i*_ into (3), it can be simplified to:

(4) $$R_{0}=\sqrt{\frac{g_{1}b_{1}^{2}}{H\left(\mu_{h}+\gamma_{h}\right)V_{1}}+\frac{g_{2}b_{2}^{2}}{H\left(\mu_{h}+\gamma_{h}\right)V_{2}}}$$

In Equations (3) and (4), *H, V*_1_, and *V*_2_ are the densities of each species at the disease‐free equilibrium point. By solving a simple system of linear equations (see Appendix S1), we have:

(5) $$V_{1}=\frac{{V_{1}}^{*}\left(1-{V_{2}}^{*}c_{12}\right)}{1-{V_{1}}^{*}{V_{2}}^{*}c_{12}c_{21}},\;V_{2}=\frac{{V_{2}}^{*}\left(1-{V_{1}}^{*}c_{21}\right)}{1-{V_{1}}^{*}{V_{2}}^{*}c_{12}c_{21}},H=\frac{\lambda_{h}}{\mu_{h}},$$

in which ${V_{i}}^{*}=\frac{\lambda_{v,i}}{\mu_{v,i}}$ gives the equilibrium vector density when there is only one vector species *i*.

Substituting Equations (2) and (5) into Equation (4), it becomes:

(6) $$R_{0}=\sqrt{\frac{1}{H\left(\mu_{h}+\gamma_{h}\right)\left(1-c_{12}c_{21}{V_{1}}^{*}{V_{2}}^{*}\right)}\left[\frac{g_{1}{\sigma_{v,1}}^{2}{V_{1}}^{*}\left(1-c_{12}{V_{2}}^{*}\right)}{{\left(1+\pi_{12}\frac{{V_{2}}^{*}\left(1-c_{21}{V_{1}}^{*}\right)}{{V_{1}}^{*}\left(1-c_{12}{V_{2}}^{*}\right)}\right)}^{2}}+\frac{g_{2}{\sigma_{v,2}}^{2}{V_{2}}^{*}\left(1-c_{21}{V_{1}}^{*}\right)}{{\left(1+\pi_{21}\frac{{V_{1}}^{*}\left(1-c_{12}{V_{2}}^{*}\right)}{{V_{2}}^{*}\left(1-c_{21}{V_{1}}^{*}\right)}\right)}^{2}}\right]}.$$

Equation (6) allows us to study the effects of feeding interference ($\pi_{\mathrm{ij}}$), interspecific competition ($c_{\mathrm{ij}}V_{j}^{*}$), and vector competence (*g*_*i*_) on disease risk.

### 2.3 Comparing disease risk between one‐ and two‐vector communities

To compare disease risk between one‐ and two‐vector communities, we compared the basic reproduction number with only one resident vector 1 ($R_{0}^{1}$) and in the presence of two vectors (*R*_0_). In the single‐vector community, the basic reproduction number $R_{0}^{1}$ can be expressed as (see Appendix S2):

(7) $${R_{0}}^{1}=\sqrt{\frac{g_{1}{b_{1}}^{2}}{H\left(\mu_{h}+\gamma_{h}\right){V_{1}}^{*}}}=\sqrt{\frac{g_{1}{\sigma_{v,1}}^{2}{V_{1}}^{*}}{H\left(\mu_{h}+\gamma_{h}\right)}}.$$

In contrast, in the two‐vector community, the formulation of the basic reproduction number *R*_0_ is given by Equation (6). Considering the complexity of Equation (6), we assumed the two vectors have the same birth ($\lambda_{v,i}=\lambda_{v}$), death rates ($\mu_{v,i}=\mu_{v}$) (i.e., ${V_{1}}^{*}={V_{2}}^{*}$), and feeding intensity ($\sigma_{v,i}=\sigma_{v}$).

If $R_{0}<{R_{0}}^{1}$, this means an increase in vector richness reduces disease risk, that is, a dilution effect occurs. Conversely, if $R_{0}>{R_{0}}^{1}$, an amplification effect occurs. $R_{0}<{R_{0}}^{1}$is equivalent to

(8) $$\left(\frac{1-c_{12}{V_{2}}^{*}}{{\left(1+\pi_{12}\frac{{V_{2}}^{*}\left(1-c_{21}{V_{1}}^{*}\right)}{{V_{1}}^{*}\left(1-c_{12}{V_{2}}^{*}\right)}\right)}^{2}}+\frac{g_{2}}{g_{1}}\frac{\left(1-c_{21}{V_{1}}^{*}\right)}{{\left(1+\pi_{21}\frac{{V_{1}}^{*}\left(1-c_{12}{V_{2}}^{*}\right)}{{V_{2}}^{*}\left(1-c_{21}{V_{1}}^{*}\right)}\right)}^{2}}\right)\frac{1}{\left(1-c_{12}c_{21}{V_{1}}^{*}{V_{2}}^{*}\right)}<1$$

Otherwise, $R_{0}>{R_{0}}^{1}$. Using the above inequality (8), we can explore how disease risk changes when the added vector species is more/less competent and superior/inferior in terms of interspecific competition and/or feeding interference.

### 2.4 Vector‐borne disease system with *N* vectors

Next, we extended the model (1) for two vectors to the case with *N* (N≥2) vectors to investigate the influence of vector richness on disease risk. The model can be described as (Takimoto et al., 2022):

(9) $$\begin{array}{c} \begin{array}{c} \frac{{\mathrm{dV}}_{S,i}}{\mathrm{dt}}=\lambda_{v,i}\left(1-\sum_{j=1,j\neq i}^{N}c_{\mathrm{ij}}V_{j}\right)-\mu_{v,i}V_{S,i}-\frac{k_{\mathrm{vh},i}b_{i}}{V_{i}}\frac{H_{I}}{H}V_{S,i}\\ \frac{{\mathrm{dV}}_{I,i}}{\mathrm{dt}}=\frac{k_{\mathrm{vh},i}b_{i}}{V_{i}}\frac{H_{I}}{H}V_{S,i}-\mu_{v,i}V_{I,i}\\ \frac{{\mathrm{dH}}_{S}}{\mathrm{dt}}=\lambda_{h}-\mu_{h}H_{S}-\sum_{i=1}^{N}\frac{k_{\mathrm{hv},i}b_{i}}{H}\frac{V_{I}}{V_{i}}H_{S}\\ \frac{{\mathrm{dH}}_{I}}{\mathrm{dt}}=\sum_{i=1}^{N}\frac{k_{\mathrm{hv},i}b_{i}}{H}\frac{V_{I}}{V_{i}}H_{S}-\mu_{h}H_{I}-\gamma_{h}H_{I}\\ \frac{{\mathrm{dH}}_{R}}{\mathrm{dt}}=\gamma_{h}H_{I}-\mu_{h}H_{R} \end{array}i=1,\ldots ,N \end{array}$$

Using the same method as described above, we obtained the community basic reproduction number $R_{0}\left(N\right)$ for *N* vector species (see Appendix S3):

(10) $$R_{0}\left(N\right)=\sqrt{\sum_{i=1}^{N}\frac{k_{\mathrm{vh},i}k_{\mathrm{hv},i}{b_{i}}^{2}}{H\left(\mu_{h}+\gamma_{h}\right)V_{i}\mu_{v,i}}}=\sqrt{\sum_{i=1}^{N}\frac{g_{i}{b_{i}}^{2}}{H\left(\mu_{h}+\gamma_{h}\right)V_{i}}}.$$

$R_{0}\left(N\right)$ here was originally derived by Takimoto et al. (2022). When there are *N* vector species, the total number of host‐vector *i* contacts *b*_*i*_ becomes:

$$b_{i}=\sigma_{v,i}V_{i}\frac{V_{i}}{V_{i}+\sum_{j=1\left(j\neq i\right)}^{N}\pi_{\mathrm{ij}}V_{j}}=\frac{\sigma_{v,i}V_{i}}{1+\sum_{j=1\left(j\neq i\right)}^{N}\pi_{\mathrm{ij}}V_{j}/V_{i}}$$

Since species may vary in their demographic and epidemiological traits, it is challenging to estimate vector‐specific trait values in multi‐vector systems. Therefore, to obtain an analytic expression connecting vector richness (*N*) to $R_{0}\left(N\right)$, we adopt the mean trait assumption that the following parameters are homogeneous across *N* vectors: the birth rate ($\lambda_{v,i}=\lambda_{v}$), the death rate ($\mu_{v,i}=\mu_{v}$), the competition coefficient ($c_{\mathrm{ij}}=c$), the intensity of feeding interference ($\pi_{\mathrm{ij}}=\pi$), the maximum feeding intensity ($\sigma_{\mathrm{vi}}=\sigma_{v}$), and the transmission probability ($k_{\mathrm{vh},i}=k_{\mathrm{vh}}$, $k_{\mathrm{hv},i}=k_{\mathrm{hv}}$) (thus $g_{i}=g$). This assumption enables us to exclude the effects of trait differences and to analytically dissect how vector richness affects disease risk. It should be noted that under the mean trait assumption, when a community contains *N* identical vectors, the effect on disease risk is not equivalent to that of a community containing only one vector but with abundance becomes *N* times greater. This can be seen by comparing the basic reproduction numbers in the two cases. The main reason for this difference is that we did not consider intraspecific competition and intraspecific feeding interference in this study.

Under the mean trait assumption, Equation (10) becomes:

(11) $$R_{0}\left(N\right)=\sqrt{N\frac{\mathrm{gb}{\left(N\right)}^{2}}{H\left(\mu_{h}+\gamma_{h}\right)V\left(N\right)}},$$

in which $V\left(N\right)$ is the equilibrium density of a given vector species:

(12) $$V\left(N\right)=\frac{V^{*}}{1+\left(N-1\right){\mathrm{cV}}^{*}},$$

Specifically, $V\left(1\right)=V^{*}=\frac{\lambda_{v}}{\mu_{v}}$.

And $b\left(N\right)$ is the expected number of host contacts for a given vector species:

(13) $$b\left(N\right)=\frac{\sigma_{v}V\left(N\right)}{1+\left(N-1\right)\pi }$$

Substituting Equations (12) and (13) into Equation (11), we obtained the basic reproduction number $R_{0}\left(N\right)$ under the mean trait assumption:

(14) $$R_{0}\left(N\right)=\sqrt{N\frac{g}{H\left(\mu_{h}+\gamma_{h}\right)}\frac{{\sigma_{v}}^{2}V^{*}}{{\left[1+\left(N-1\right)\pi \right]}^{2}\left[1+\left(N-1\right){\mathrm{cV}}^{*}\right]}}.$$

All simulations were performed using MATLAB. The main source code is available in Data S1.

## 3 RESULTS

### 3.1 Effects of interspecific competition, feeding interference, and vector competence on disease risk in the two‐vector community

**FIGURE 1:**
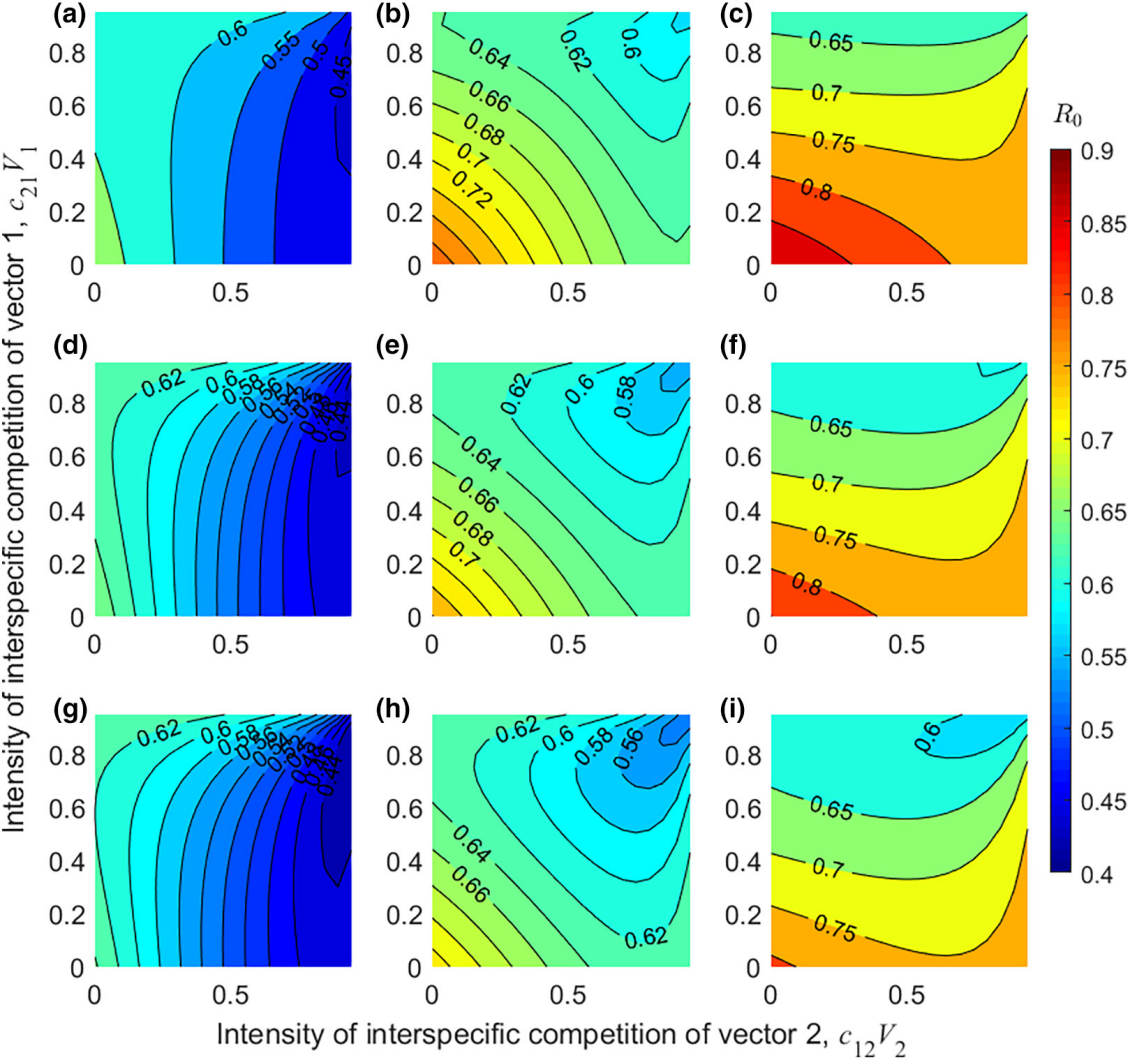
The impact of interspecific competition ($c_{12}V_{2}$ and$c_{21}V_{1}$) on $R_{0}$. Different colors represent different values of $R_{0}$. Rows represent $\pi_{12}/\pi_{21}$= 0.5 (a–c), 1 (d–f), and 2 (g–i) from top to bottom, and columns represent $g_{1}/g_{2}$ = 0.5 (a, d, g), 1 (b, e, h), and 2 (c, f, i) from left to right. Other parameters:$\mu_{h}=0.2$, $\gamma_{h}=0.1$, H=50, $V_{1}=V_{2}=500$,$\sigma_{v,1}=\sigma_{v,2}=0.25$, $\pi_{21}=0.2$, and $g_{2}=0.2$.

As seen in each subplot of Figure 1, disease risk was higher when the interspecific competition of the two vector species was low. As the interspecific competition of the two vector species increased, the risk of disease gradually decreased. By comparing the three subplots in each row, we found that when two vectors had the same competence (i.e., $g_{1}/g_{2}=1$), disease risk decreased at the same rate as the competitiveness of the two vectors increased (the subplots are symmetric). In contrast, when two vectors had different competence, disease risk decreased more rapidly as the interspecific competition intensity of the higher competence vector increased. For example, when vector 2 was more competent than vector 1 (i.e., $g_{1}/g_{2}=0.5$), disease risk decreased more rapidly as the interspecific competition intensity of vector 2 ($c_{12}V_{2}$) increased, and vice versa (i.e.,$g_{1}/g_{2}=2$). Furthermore, a comparison of the three subplots in each column revealed that, regardless of how changes in feeding interference, there was little impact on the effect of interspecific competition on disease risk.

**FIGURE 2:**
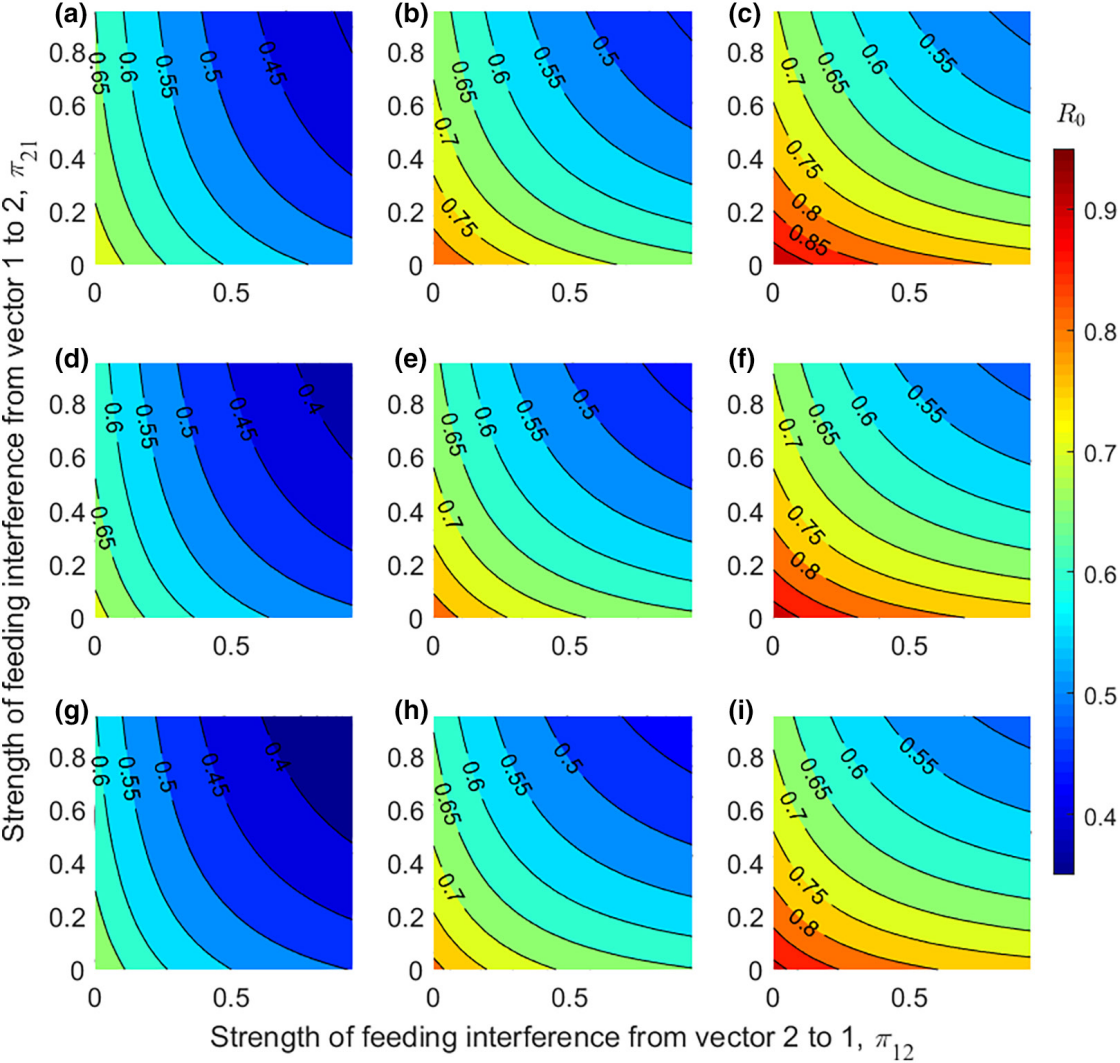
The effect of feeding interference ($\pi_{12}$ and $\pi_{21}$) on $R_{0}$. Rows represent $c_{21}V_{1}/c_{12}V_{2}$= 0.5 (a–c), 1 (d–f), and 2 (g–i) from top to bottom, and columns represent $g_{1}/g_{2}$ = 0.5 (a, d, g), 1 (b, e, h), and 2 (c, f, i) from left to right. Here, $c_{12}V_{2}=0.2$ and the values of other parameters are the same as those in Figure 1.

Figure 2 illustrates how disease risk related to feeding interference ($\pi_{12}$ and $\pi_{21}$) and how this relationship varied with vector competence and interspecific competition. It was found that disease risk decreased with the increase of feeding interference, with disease risk being highest when feeding interferences of the two vectors were minimal (bottom left of each subplot), and lowest when feeding interferences of the two vectors were maximal (top right of each subplot). In addition, disease risk decreased at the same rate as feeding interferences of both vectors increased, regardless of whether vector 1 is more/less competent than vector 2 or whether vector 1 is superior/inferior competitive than vector 2.

### 3.2 Dilution effects versus amplification effects

In Section 2.3, we calculated the basic reproduction numbers for the single‐vector model ($R_{0}^{1}$) and the two‐vector model (*R*_0_) and obtained the threshold conditions for the occurrence of the dilution and amplification effects. It was found that increases in vector richness could result in both amplification and dilution effects, depending on the strength of interspecific competition, feeding interference, and competence of the added vector species relative to the focal vector species.

**FIGURE 3:**
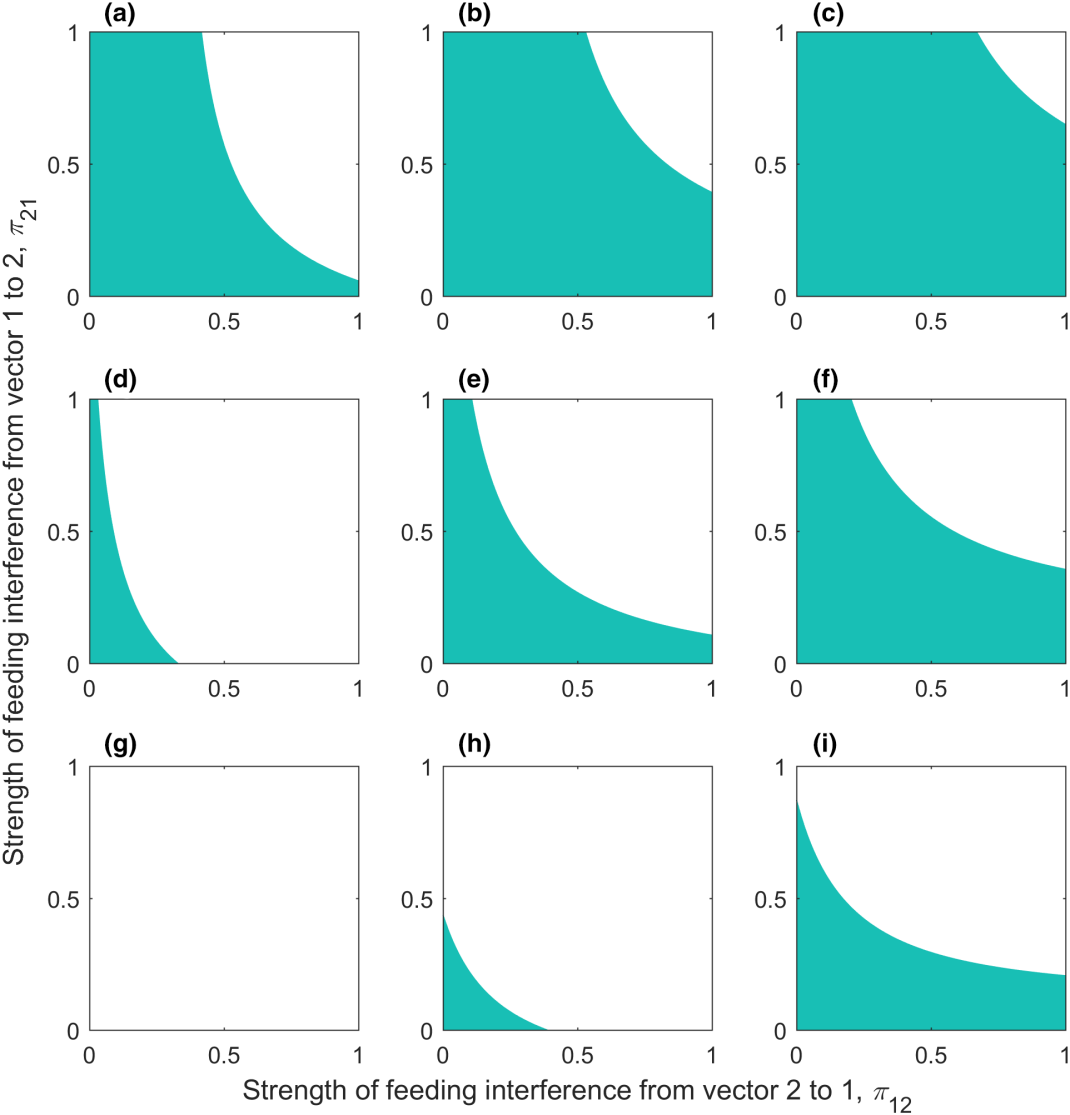
Parameter space for amplification and dilution effects. The green shaded area represents where amplification effects occur, while the unshaded (white) area represents where dilution effects occur. Rows represent $c_{12}/c_{21}$= 0.5 (a–c), 1 (d–f), and 1.5 (g–i) from top to bottom, and columns represent $g_{2}/g_{1}$ = 0.5 (a, d, g), 1 (b, e, h), and 1.5 (c, f, i) from left to right. Here $c_{21}V_{1}=0.4$.

In Figure 3, the three columns indicate that the competence of the added vector is less than, equal to, and greater than the resident vector 1, and the three rows indicate that the competitive intensity of the added vectors is less than, equal to, and greater than the resident vector 1. Green shaded areas are areas of amplification effects (${R_{0}>R}_{0}^{1}$) and unshaded areas are areas of dilution effects (${R_{0}<R}_{0}^{1}$). Firstly, as seen in each subplot, increasing vector richness tended to result in an amplification effect when feeding interference was low. In contrast, as the intensity of feeding interference increased, it shifted to a dilution effect. Secondly, for a fixed ratio of interspecific competition, the size of the region in which dilution or amplification effect occurs was closely related to the competence of the added vector (Figure 3). When a low‐competence vector was added to the community (i.e., $g_{2}/g_{1}=0.5$), the shaded area became larger, that is, the dilution effect was more prone to occur. In contrast, when a high‐competence vector was added to the community (i.e., $g_{2}/g_{1}=1.5$), the area for an amplification effect became larger. This means the positive effects of adding a high‐competence vector species on pathogen transmission outweighed the negative effects of vector interference, thus favoring the occurrence of amplification effects. Similarly, for a fixed ratio of vector competence, the more competitive the added vector was, the smaller the area in which the amplification effect occurs. When the added vector was of low competence and highly competitive, only dilution effects may occur (Figure 3g). This means the addition of a highly competitive vector species may exacerbate the negative effects of vector interference, thus favoring the occurrence of dilution effects.

### 3.3 Effect of vector richness on disease risk in communities with *N* vectors

From the expression of *R*_0_(*N*), it can be seen that vector richness can both amplify and dilute disease risk: when vector richness (*N*) increases, pathogen transmission also increases (since *R*_0_(*N*) is a sum of *N* terms in Equation [10]), leading to an amplification effect. However, higher vector richness also leads to greater competition and feeding interference, leading to a decrease in the number of host–vector contacts *b*(*N*) and a subsequent dilution effect. Therefore, the overall effect of vector richness on disease risk depends on the relative magnitude of the dilution and amplification effects.

**FIGURE 4:**
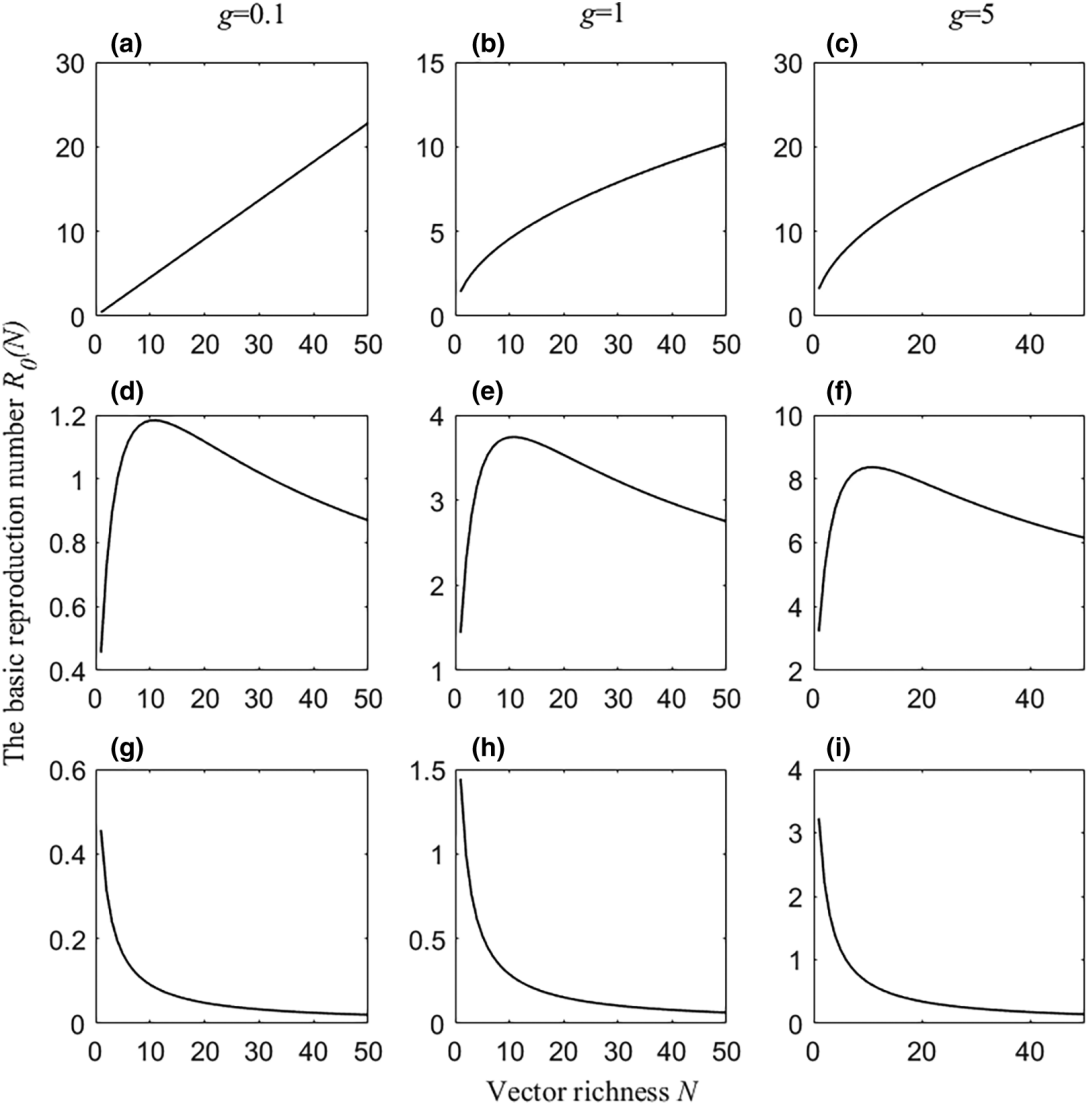
The impact of vector richness (*N*) on community $R_{0}\left(N\right)$ for different values of vector competence (*g*). Columns represent g = 0.1 (a, d, g), 1 (b, e, h), and 5 (c, f, i) from left to right. The first row represent ${\mathrm{cV}}^{*}$=0 and π=0(a–c). The second row represent ${\mathrm{cV}}^{*}$=0.1 and π=0.2 (d–f). The third row represent ${\mathrm{cV}}^{*}$=0.3 and π=0.8 (g–i). Other parameters include:$\mu_{h}=0.2$, $\gamma_{h}=0.1$, H=50, V=500, and σ=0.25.

On the one hand, for a certain vector competence, *R*_0_(*N*) showed three patterns with vector richness: when interspecific competition and feeding interference were zero, *R*_0_(*N*) increased with vector richness, indicating an amplification effect (Figure 4a–c). For moderate values of interspecific competition and feeding interference, *R*_0_(*N*) first increased and then decreased with vector richness (i.e., both amplification and dilution effects occurred) (Figure 4d–f). When interspecific competition and feeding interference were high, *R*_0_(*N*) decreased with vector richness, indicating a dilution effect (Figure 4g–i). Figures S1 and S2 in Appendix S4 showed the effect of feeding interference or interspecific competition on the vector richness–disease risk relationship, respectively. The findings in Figure 4 are consistent with those in Figures S1 and S2.

On the other hand, the higher the vector competence, the higher the disease risk. However, the trend in vector richness‐disease risk relationship was unchanged regardless of changes in vector competence, providing that intraspecific competition and feeding interference remained constant. This suggests that when all vectors were homogeneous, the relationship between vector richness and disease risk was driven by the strength of feeding interference and interspecific competition, and changes in vector competence only quantitatively but not qualitatively altered the vector richness–disease risk relationship.

### 3.4 Global sensitivity analysis

**FIGURE 5:**
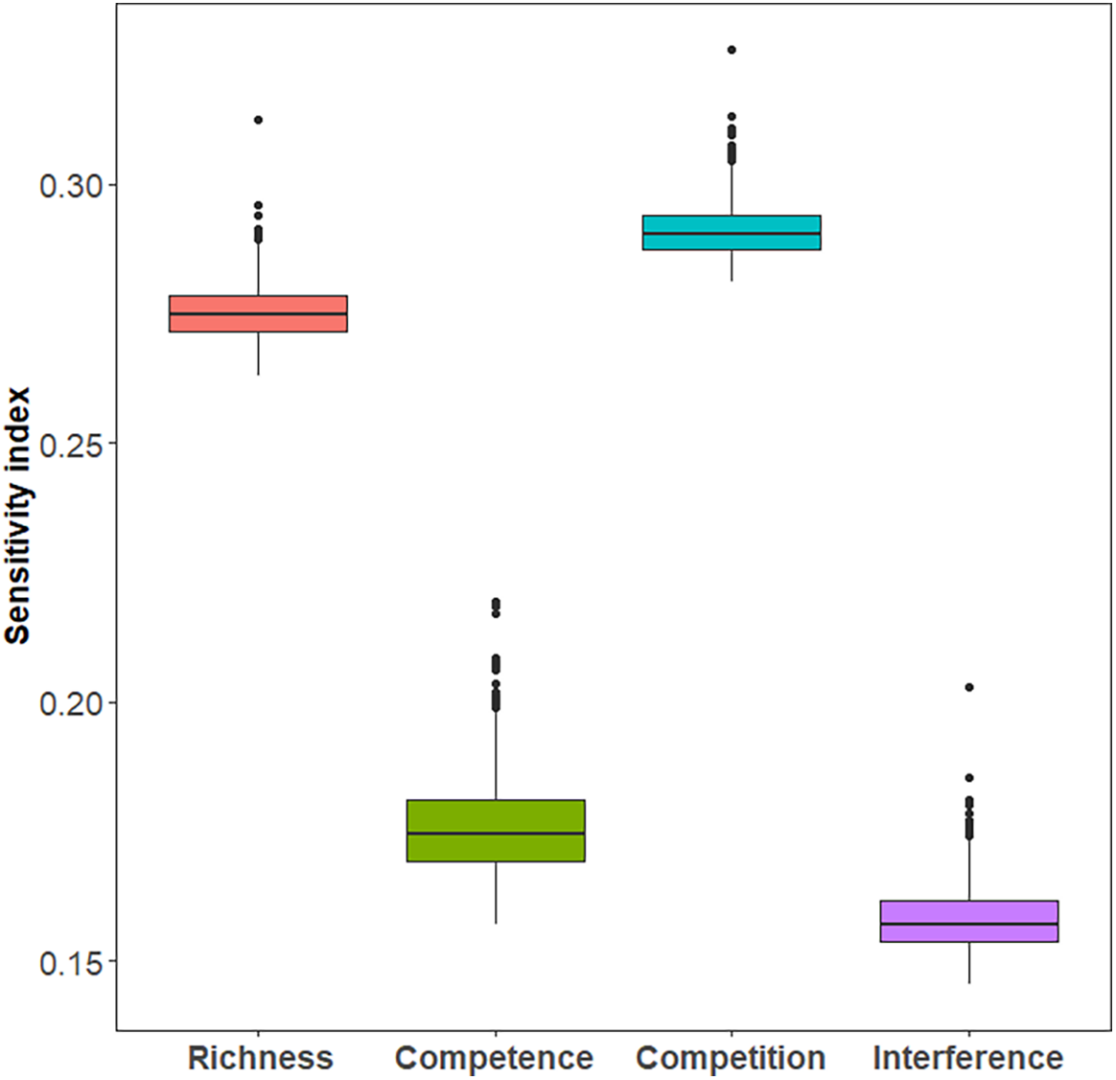
Sensitivity indices of the parameters on $R_{0}\left(N\right)$. The boxes represent the 95% bootstrap confidence intervals and the black lines represent the mean of the indices. Other parameters include:$\mu_{h}=0.2$, $\gamma_{h}=0.1$, H=50, V=500, and σ=0.25.

To assess the effect of parameters on *R*_0_(*N*), we performed a global sensitivity analysis. We used the open‐source *R* package called SAFER to calculate the sensitivity indices (https://safetoolbox.github.io/) (Noacco et al., 2019). Global sensitivity analyses showed that vector richness and interspecific competition were the most influential parameters on *R*_0_(*N*), followed by vector competence and feeding interference (Figure 5).

## 4 DISCUSSION

A central goal in disease ecology is to identify the factors that drives the spread of infectious diseases. Vectors are important intermediate hosts for many parasites and greatly impact parasite transmission (Hoi et al., 2022), yet the role of vector richness and vector competence in moderating the diversity–disease relationship has received little attention. Based on the theoretical model of Takimoto et al. (2022), we examined how the combined effect of vector richness, vector competence, and vector interspecific interactions impact disease risk in a multi‐vector community. It was found that disease risk declined more rapidly as interspecific competition of the high‐competence vector increased. When vector richness increases, the positive effects of adding a high‐competence vector species on disease transmission may outweigh the negative effects of feeding interference due to increased vector richness, making an amplification effect more likely to occur. While the addition of a highly competitive vector species may exacerbate the negative effects of feeding interference, making a dilution effect more likely to occur. In the *N*‐vector model, when all vectors were homogeneous, the effect of increased vector richness on disease risk was driven by the strength of feeding interference and interspecific competition, and changes in vector competence only quantitatively but not qualitatively altered the vector richness–disease risk relationship.

Increased vector richness may have a complex impact on disease risk. On the one hand, higher vector richness can prompt disease risk by increasing pathogen transmission routes (Hoi et al., 2020) and/or total vector abundance in the community (Takimoto et al., 2022; Tilman et al., 1996). On the other hand, higher vector richness may increase interspecific competition and feeding interference among vectors, thereby inhibiting pathogen transmission (Figures 1 and 2). It is also related to the competence and interspecific competition of the vectors added to the community (Figure 3). The overall effect of vector richness on disease risk depends on the trade‐off between positive and negative effects, with an amplification effect occurring if the positive effect outweighs the negative effect, and vice versa for a dilution effect. This may explain why previous studies (McMillan et al., 2019; Park et al., 2015; Roche et al., 2013) have often shown amplification effects, possibly because for many real‐world vector‐borne diseases, feeding interference and interspecific competition among vectors are weaker, leading to the dominance of positive effects.

Empirical studies have also confirmed that amplification effects occur when interspecific interactions among vectors are weak (Hoi et al., 2020; Park et al., 2015), consistent with our findings. In addition, our results suggest, at least in theory, that a dilution effect occurs if feeding interference and/or interspecific competition are strong. Although this phenomenon has not been verified in natural communities, the theoretical results suggest that dilution effects are likely to occur. It also suggests that it may be inaccurate to assume that increased vector richness will only increase the spread of pathogens, and that vector competition and feeding interference should be taken into account in future empirical studies.

Considering the role of vector competence in examining the vector richness–disease risk relationship is a unique contribution of this study. We found whether vector richness increases or decreases disease risk is closely related to vector competence. In fact, in some natural communities, vector competence is negatively correlated with vector richness (Hoi et al., 2020). For example, *Anopheles gambiae*, a key vector for malaria, is present in almost all communities within its range, but dominates in communities with low mosquito richness; meanwhile, other vectors, such as *Anopheles funestus*, appear only in communities with high vector richness (Hoi et al., 2020). Likewise, *Aedes aegypti*, a major vector for chikungunya, dengue, and yellow fever, was found in greater abundance in rural and urban areas with lower species richness than in forests with higher species richness in central Thailand (Thongsripong et al., 2013). If species‐poor communities tend to include a high proportion of high‐competence vectors, dilution or mixed amplification‐dilution effects may be more common than amplification effects. Conversely, the addition of high‐competence vectors to a community may shift dilution effects to amplification effects (Figure 3). These results are in line with findings of Hoi et al. (2020) that the influence of vector richness on disease risk depended on the covariation between the order of addition of vector species and vector competence. Our theoretical results emphasize the importance of incorporating vector competence into studies of the vector richness–disease risk relationship. However, empirical results in this area are scarce and more empirical studies are needed in the future to validate our theoretical results.

This study makes an important contribution in linking vector richness, vector competence, and interspecific interactions to understand their joint impact on disease risk, an aspect of disease ecology that has been neglected in previous studies. In order to obtain analytic results, a number of simplified assumptions were made. For example, we did not consider intraspecific competition in vectors, which plays an important role in balancing populations. We only considered the case where a community contains one host species, but many vector‐borne diseases may involve different vectors and different hosts (Pessanha et al., 2011; Tanga et al., 2011). Moreover, when studying the vector‐borne model containing *N* vector species, we assumed that all vectors were homogeneous. This assumption is difficult to hold in real‐world communities (Cator et al., 2020). Despite these limitations, this work clarifies the role of vector competence in the relationship between vector richness and disease risk and provides a new perspective for studying the diversity–disease relationship. It also provides theoretical guidance for vector management and disease prevention strategies.

## AUTHOR CONTRIBUTIONS

**Lifan Chen:** Conceptualization (lead); formal analysis (lead); methodology (lead); software (lead); writing – original draft (lead); writing – review and editing (lead). **Zhiying Tan:** Formal analysis (equal); methodology (equal); writing – original draft (equal). **Ping Kong:** Software (equal); validation (equal); writing – original draft (equal). **Yanli Zhou:** Software (equal); validation (equal); writing – original draft (equal). **Liang Zhou:** Validation (equal); writing – original draft (equal); writing – review and editing (equal).

## FUNDING INFORMATION

This work was supported by NSFC (31700466), Foundation of Shanghai University of Medicine and Health Sciences, and Climb Plan of Shanghai University of Medicine and Health Sciences.

## Supporting information


Appendix S1

Data S1

## Data Availability

Matlab code generating Figures 1, 2, 3, 4 is provided online as electronic Data S1.
