# Supplementary material for: Impact of vector richness on the risk of vector‐borne disease: The role of vector competence
Source: Ecol Evol. 2024 Mar 1;14(3):e11082. doi: 10.1002/ece3.11082 (PMC10905232; doi:10.1002/ece3.11082)
Supplement: Supplementary file 1 — Appendix S1 [file ECE3-14-e11082-s001.docx]

**Appendix S1.**

We calculated the basic reproduction number of equation (1) in the main text using the dominant eigenvalue of the next-generation matrix. To this end, we linearized the infectious subsystem of equation (1) at the disease-free equilibrium (*H,* $V_{1}$, and $V_{2}$):

$\begin{matrix} \frac{dV_{I,1}}{dt}=\frac{k_{vh,1}b_{1}H_{I}}{H}-\mu_{V,1}V_{I,1} \\ \frac{dV_{I,2}}{dt}=\frac{k_{vh,2}b_{2}H_{I}}{H}-\mu_{V,2}V_{I,2} \\ \frac{dH_{I}}{dt}=\sum_{i=1}^{2} \frac{k_{hv,i}b_{i}V_{I,i}}{V_{i}}-\mu_{h}H_{I}-\gamma_{h}H_{I} \end{matrix}$ (S1)

The transmission matrix *F* which accounts for the rate of new infections is derived as:

$F=\left(\begin{matrix} 0 & 0 & \frac{k_{vh,1}b_{1}}{H} \\ 0 & 0 & \frac{k_{vh,2}b_{2}}{H} \\ \frac{k_{hv,1}b_{1}}{V_{1}} & \frac{k_{hv,2}b_{2}}{V_{2}} & 0 \end{matrix}\right)$.

The transition matrix *V* is derived as:

$V=\left(\begin{matrix} -\mu_{v,1} & 0 & 0 \\ 0 & -\mu_{v,2} & 0 \\ 0 & 0 & -(\mu_{h}+\gamma_{h}) \end{matrix}\right)$.

Therefore, the next-generation matrix *K* is:

$K=-FV^{-1}=\left(\begin{matrix} 0 & 0 & \frac{k_{vh,1}b_{1}}{H(\mu_{h}+\gamma_{h})} \\ 0 & 0 & \frac{k_{vh,2}b_{2}}{H(\mu_{h}+\gamma_{h})} \\ \frac{k_{hv,1}b_{1}}{V_{1}\mu_{v,1}} & \frac{k_{hv,2}b_{2}}{V_{2}\mu_{v,2}} & 0 \end{matrix}\right)$.

To obtain the basic reproduction number$R_{0}$, we solved the dominant eigenvalue of *K*, i.e., $\left|K-R_{0}E\right|=0$. It can be written as:

$\left|K-R_{0}E\right|=\left(\begin{matrix} -R_{0} & 0 & \frac{k_{vh,1}b_{1}}{H(\mu_{h}+\gamma_{h})} \\ 0 & -R_{0} & \frac{k_{vh,2}b_{2}}{H(\mu_{h}+\gamma_{h})} \\ \frac{k_{hv,1}b_{1}}{V_{1}\mu_{v,1}} & \frac{k_{hv,2}b_{2}}{V_{2}\mu_{v,2}} & -R_{0} \end{matrix}\right)=\left(\begin{matrix} -R_{0} & 0 & \frac{k_{vh,1}b_{1}}{H(\mu_{h}+\gamma_{h})} \\ 0 & -R_{0} & \frac{k_{vh,2}b_{2}}{H(\mu_{h}+\gamma_{h})} \\ 0 & \frac{k_{hv,2}b_{2}}{V_{2}\mu_{v,2}} & -R_{0}+\frac{\frac{k_{hv,1}b_{1}}{V_{1}\mu_{v,1}}\frac{k_{vh,1}b_{1}}{H(\mu_{h}+\gamma_{h})}}{R_{0}} \end{matrix}\right)=0\text{，}$then we obtained the expression of $R_{0}$ (equation (3) in the main text):

$R_{0}=\sqrt{\frac{k_{vh,1}k_{hv,1}b_{1}^{2}}{H(\mu_{h}+\gamma_{h})V_{1}\mu_{v,1}}+\frac{k_{vh,2}k_{hv,2}b_{2}^{2}}{H(\mu_{h}+\gamma_{h})V_{2}\mu_{v,2}}}$. (S2)

In equation (S2), *H,* $V_{1}$, and $V_{2}$ are the equilibrium densities of the host and the vectors. To find the values of *H,* $V_{1}$, and $V_{2}$, we solved the following system of linear equations:

$\begin{matrix} \frac{dV_{1}}{dt}=\lambda_{v,1}(1-c_{12}V_{2})-\mu_{v,1}V_{1} \\ \frac{dV_{2}}{dt}=\lambda_{v,2}(1-c_{21}V_{1})-\mu_{v,2}V_{2} \\ \frac{dH}{dt}=\lambda_{h}-\mu_{h}H \end{matrix}$ (S3)

The first and second equations are derived by adding the left and right sides of the equations for susceptible and infected vector densities in equation (1) (in the main text). The third equation is derived similarly for susceptible, infected, and recovered host densities in equation (1). Letting the right-hand side of each equation in equation (S3) be zero, we obtained:

$H=\frac{\lambda_{h}}{\mu_{h}}$，

$\begin{matrix} V_{1}=\frac{\lambda_{v,1}\mu_{v,2}-\lambda_{v,1}\lambda_{v,2}c_{12}}{\mu_{v,1}\mu_{v,2}-\lambda_{v,1}\lambda_{v,2}c_{12}c_{21}}=\frac{V_{1}^{*}(1-V_{2}^{*}c_{12})}{1-V_{1}^{*}V_{2}^{*}c_{12}c_{21}} \\ V_{2}=\frac{\lambda_{v,2}\mu_{v,1}-\lambda_{v,1}\lambda_{v,2}c_{21}}{\mu_{v,1}\mu_{v,2}-\lambda_{v,1}\lambda_{v,2}c_{12}c_{21}}=\frac{V_{2}^{*}(1-V_{1}^{*}c_{21})}{1-V_{1}^{*}V_{2}^{*}c_{12}c_{21}} \end{matrix}$ ,

where $V_{i}^{*}=\frac{\lambda_{v,i}}{\mu_{v,i}}$ gives the equilibrium vector density when there is only one vector species *i*.

**Appendix S2.**

In this section, we calculated the basic reproduction number $R_{0}^{1}$ with only one resident vector 1. Similar to Appendix S1, we linearized the infectious subsystem at the disease-free equilibrium (*H,*$V^{*}$):

$$\frac{dV_{I,1}}{dt}=\frac{k_{vh,1}b_{1}H_{I}}{H}-\mu_{v,1}V_{I,1}$$

$$\frac{dH_{I}}{dt}=\frac{k_{hv\text{，}1}b_{1}V_{I\text{，}1}}{V_{1}}-\mu_{h}H_{I}-\gamma_{h}H_{I}$$

Then the transmission matrix *F* is:

$F=\left(\begin{matrix} 0 & \frac{k_{vh,1}b_{1}}{H} \\ \frac{k_{hv,1}b_{1}}{V^{*}} & 0 \end{matrix}\right)$.

The transition matrix *V* is:

$V=\left(\begin{matrix} -\mu_{v} & 0 \\ 0 & -(\mu_{h}+\gamma_{h}) \end{matrix}\right)$.

Therefore, the next-generation matrix *K* is:

$K=-FV^{-1}=\left(\begin{matrix} 0 & \frac{k_{vh,1}b_{1}}{H(\mu_{h}+\gamma_{h})} \\ \frac{k_{hv,1}b_{1}}{V_{1}^{*}\mu_{v}} & 0 \end{matrix}\right)$.

The dominant eigenvalue of *K* is the basic reproduction number$R_{0}^{1}$,

$R_{0}^{1}=\sqrt{\frac{k_{vh,1}k_{hv,1}b_{1}^{2}}{H(\mu_{h}+\gamma_{h})V_{1}^{*}\mu_{v,1}}}=\sqrt{\frac{g_{1}b_{1}^{2}}{H(\mu_{h}+\gamma_{h})V_{1}^{*}}}$.

**Appendix S3.**

Using the same method as in Appendix S1, we linearized the infectious subsystem of equation (9) at the disease-free equilibrium (*H,* $V_{1}$, $V_{2}$ ,…, $V_{N}$):

$\begin{matrix} \frac{dV_{I,i}}{dt}=\frac{k_{vh,i}b_{i}H_{I}}{H}-\mu_{v,i}V_{I,i} \\ \frac{dH_{I}}{dt}=\sum_{i=1}^{N} \frac{k_{hv,i}b_{i}V_{I,i}}{V_{i}}-\mu_{h}H_{I}-\gamma_{h}H_{I} \end{matrix}$ ，*i=1,…,N*

Then the transmission matrix *F* is

$F=\left(\begin{matrix} 0 & 0 & \cdots& \frac{k_{vh,1}b_{1}}{H} \\ 0 & 0 & \cdots& \frac{k_{vh,2}b_{2}}{H} \\ \vdots& \vdots& \ddots& \vdots\\ \frac{k_{hv,1}b_{1}}{V_{1}} & \frac{k_{hv,2}b_{2}}{V_{2}} & \cdots& 0 \end{matrix}\right)$.

The transition matrix *V* is is derived as

$V=\left(\begin{matrix} -\mu_{v,1} & 0 & \cdots& 0 \\ 0 & -\mu_{v,2} & \cdots& 0 \\ \vdots& \vdots& \ddots& \vdots\\ 0 & 0 & \cdots& -(\mu_{h}+\gamma_{h}) \end{matrix}\right)$.

Therefore, the next-generation matrix *K* is,

$K=-FV^{-1}=\left(\begin{matrix} 0 & 0 & \cdots& \frac{k_{vh,1}b_{1}}{H(\mu_{h}+\gamma_{h})} \\ 0 & 0 & \cdots& \frac{k_{vh,2}b_{2}}{H(\mu_{h}+\gamma_{h})} \\ \vdots& \vdots& \ddots& \vdots\\ \frac{k_{hv,1}b_{1}}{V_{1}\mu_{v,1}} & \frac{k_{hv,2}b_{2}}{V_{2}\mu_{v,2}} & \cdots& 0 \end{matrix}\right)$.

To calculate the basic reproduction number$R_{0}$, we solved the characteristic equation$\left|K-R_{0}E\right|=0$( 𝐸 is the identity matrix),

$\begin{matrix} \left|K-R_{0}E\right|=\left(\begin{matrix} -R_{0} & 0 & \cdots& \frac{k_{vh,1}b_{1}}{H(\mu_{h}+\gamma_{h})} \\ 0 & -R_{0} & \cdots& \frac{k_{vh,2}b_{2}}{H(\mu_{h}+\gamma_{h})} \\ \vdots& \vdots& \ddots& \vdots\\ \frac{k_{hv,1}b_{1}}{V_{1}\mu_{v,1}} & \frac{k_{hv,2}b_{2}}{V_{2}\mu_{v,2}} & \cdots& -R_{0} \end{matrix}\right) \\ \text{ }\mathop{=}\limits^{r_{N}+\frac{\frac{k_{hv,1}b_{1}}{V_{1}\mu_{v,1}}}{R_{0}}r_{1}} \left(\begin{matrix} -R_{0} & 0 & \cdots& \frac{k_{vh,1}b_{1}}{H(\mu_{h}+\gamma_{h})} \\ 0 & -R_{0} & \cdots& \frac{k_{vh,2}b_{2}}{H(\mu_{h}+\gamma_{h})} \\ \vdots& \vdots& \ddots& \vdots\\ 0 & \frac{k_{hv,2}b_{2}}{V_{2}\mu_{v,2}} & \cdots& -R_{0}+\frac{\frac{k_{vh,1}k_{hv,1}b_{1}^{2}}{H(\mu_{h}+\gamma_{h})V_{1}\mu_{v,1}}}{R_{0}} \end{matrix}\right) \\ \text{ }=(-R_{0})\left(\begin{matrix} -R_{0} & \cdots& \frac{k_{vh,2}b_{2}}{H(\mu_{h}+\gamma_{h})} \\ \vdots& \ddots& \vdots\\ \frac{k_{hv,2}b_{2}}{V_{2}\mu_{v,2}} & \cdots& -R_{0}+\frac{\frac{k_{vh,1}k_{hv,1}b_{1}^{2}}{H(\mu_{h}+\gamma_{h})V_{1}\mu_{v,1}}}{R_{0}} \end{matrix}\right) \end{matrix}$.

Repeating the above process, we have

$\left|K-R_{0}E\right|=(-R_{0})^{N}[-R_{0}+\sum_{i=1}^{N} \frac{\frac{k_{vh,i}k_{hv,i}b_{i}^{2}}{H(\mu_{h}+\gamma_{h})V_{i}\mu_{v,i}}}{R_{0}}]=(-R_{0})^{N-1}[R_{0}^{2}-\sum_{i=1}^{N} \frac{k_{vh,i}k_{hv,i}b_{i}^{2}}{H(\mu_{h}+\gamma_{h})V_{i}\mu_{v,i}}]$.

Letting $\left|K-R_{0}E\right|=0$, we can obtain (equation (10)):

$R_{0}=\sqrt{\sum_{i=1}^{N} \frac{k_{vh,i}k_{hv,i}b_{i}^{2}}{H(\mu_{h}+\gamma_{h})V_{i}\mu_{v,i}}}$.

Note that$R_{0}(N)$ here was originally derived by Takimoto et al. (2022).

Under the mean-trait assumption, the equilibrium vector densities $V_{i}$(*i*=1,…, *N*) are the same, therefore we use $V(N)$ to denote the equilibrium density of a given vector species. To obtain the expressions for $V(N)$ and *H* (the equilibrium host density), we added the left and right sides of the equations in system (9) for the vector density and the host density to obtain:

$\begin{matrix} \frac{dV_{i}}{dt}=\lambda_{v}\left(1-\sum_{j=1,j\neq i}^{n} cV_{j}\right)-\mu_{v}V_{i} \\ \frac{dH}{dt}=\lambda_{h}-\mu_{h}H \end{matrix}$ (S4)

Letting the right-hand side of each equation in equation (S4) be zero, we obtained:

$H=\frac{\lambda_{h}}{\mu_{h}}$，

$V_{i}=\frac{V^{*}}{1+(n-1)cV^{*}}$,

in which $V^{*}=\frac{\lambda_{v}}{\mu_{v}}$ gives the equilibrium vector density when there is only one vector species.

**Appendix S4: Figures**

**Figure S1**

**
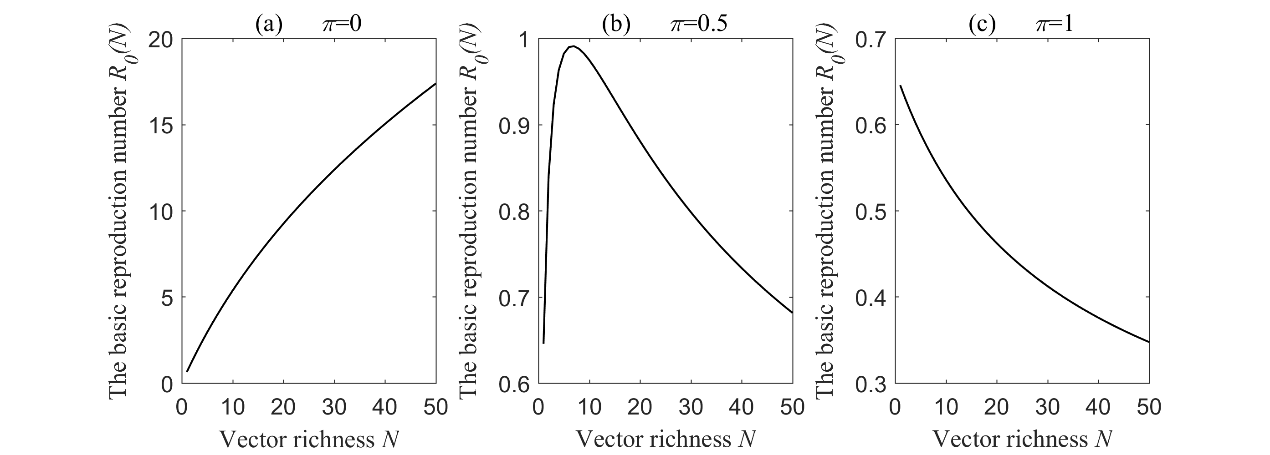
**

**Figure S1.** The impact of vector richness (*N*) on community $R_{0}(N)$ for different values of feeding interference ($\pi$). From left to right, $\pi$ is (a) 0, (b) 0.5, and (c) 1. Other parameters include: $\mu_{h}=0.2$, $\gamma_{h}=0.1$, $H=50$, $V=500$, $g=0.2$, $\sigma=0.25$, and $cV^{*}=0.05$.

Figure S1 shows the relationship between vector richness (*N*) and community disease risk ($R_{0}(N)$), as well as how this relationship was moderated by feeding interference ($\pi$). Depending on the intensity of feeding interference, $R_{0}(N)$ showed three distinct patterns with vector richness: monotonic increasing, monotonic decreasing, and hump-shaped. When there was no feeding interference (Fig. S1(a)), $R_{0}(N)$ increased with vector richness (i.e., an amplification effect occurred). With low levels of feeding interference (Fig. S1 (b)), $R_{0}(N)$ first increased and then decreased with vector richness (i.e., both amplification and dilution effects occurred). With high levels of feeding interference (Fig. S1(c)), $R_{0}(N)$ decreased with vector richness (i.e., a dilution effect occurred).

From Fig. S1, we also found feeding interference could affect the probability of disease outbreaks and disease prevalence. When $\pi$=0 (Fig. S1(a)), $R_{0}(N)$>1 held for all *N* > 3, meaning that a disease would become endemic. However, for$\pi$=0.5 or 1 (Figs. S1(b)and S1(c)), $R_{0}(N)$<1 and the disease would eventually die out.

**Figure S2**


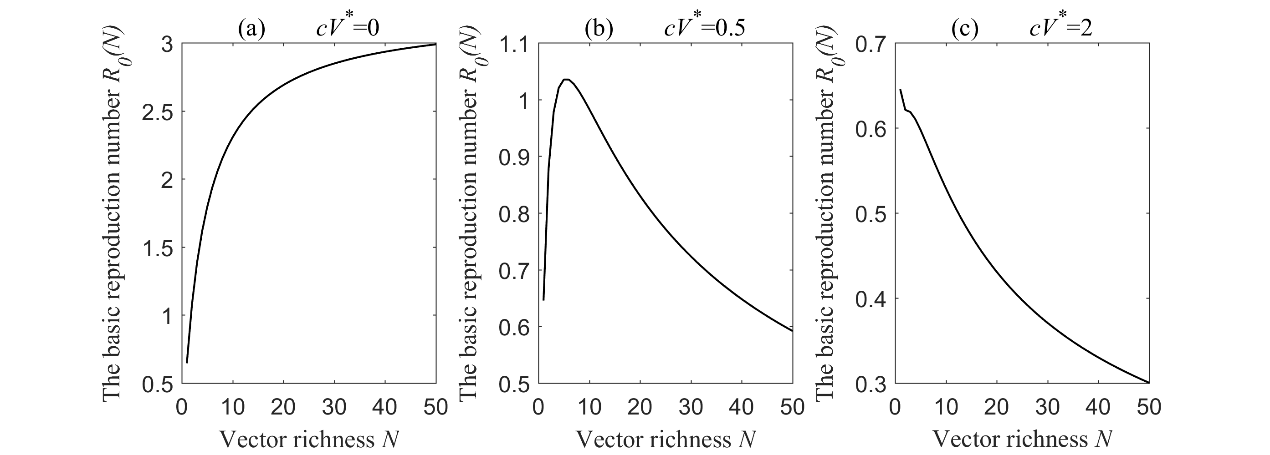


**Figure S2.** The impact of vector richness (*N*) on community $R_{0}(N)$ for different values of interspecific competition ($cV^{*}$). From left to right, $cV^{*}$ is (a) 0, (b) 0.5, and (c) 2. Other parameters include:$\mu_{h}=0.2$, $\gamma_{h}=0.1$, $H=50$, $V=500$, $g=0.2$, $\sigma=0.25$, and $\pi=0.2$.

Figure S2 illustrates the impact of vector competition ($cV^{*}$) on the relationship between vector richness and disease risk ($R_{0}(N)$). A similar pattern was observed as for feeding interference (see Fig. S1). When there was no interspecific competition among vectors, $R_{0}(N)$ increased monotonically with vector richness (Fig. S2(a)). When there was moderate interspecific competition, $R_{0}(N)$ first increased and then decreased with vector richness, showing a hump-shaped pattern (Fig. S2(b)). When interspecific competition was high, $R_{0}(N)$ decreased monotonically with vector richness (Fig. S2 (c)).
